# A Study of eHealth from the Perspective of Social Sciences

**DOI:** 10.3390/healthcare9020108

**Published:** 2021-01-21

**Authors:** Juan Uribe-Toril, José Luis Ruiz-Real, Bruno José Nievas-Soriano

**Affiliations:** 1 Faculty of Economics and Business, University of Almeria, 04120 Almería, Spain; juribe@ual.es; 2 Nursing, Physiotherapy and Medicine Department, University of Almería, 04120 Almería, Spain; brunonievas73@gmail.com

**Keywords:** eHealth, mHealth, telemedicine, telehealth, social sciences, bibliometrics

## Abstract

The field of social sciences has become increasingly important in eHealth. Patients currently engage more proactively with health services. This means that eHealth is linked to many different areas of Social Sciences. The main purpose of this research is to analyze the state-of-the-art research on eHealth from the perspective of social sciences. To this end, a bibliometric analysis was conducted using the Web of Science database. The main findings show the evolution of publications, the most influential countries, the most relevant journals and papers, and the importance of the different areas of knowledge. Although there are some studies on eHealth within social sciences, most of them focus on very specific aspects and do not develop a holistic analysis. Thus, this paper contributes to academia by analyzing the state-of-the-art of research, as well as identifying the most relevant trends and proposing future lines of research such as the potential of eHealth as a professional training instrument, development of predictive models in eHealth, analysis of the eHealth technology acceptance model (TAM), efficient integration of eHealth within public systems, efficient budget management, or improvement in the quality of service for patients.

## 1. Introduction

The Internet is a phenomenon that no one could have predicted [1]. It has changed the way we access and use the information [2]. A few years ago, textbooks were the only source of medical information. Nowadays, anyone can find medical information by accessing the Internet from almost anywhere in the world [3]. As a consequence, people have changed the way they search for information and make decisions about their health [4]. The interest of people in the Internet as a tool for searching for health information is rising rapidly and online searches about health have increased in recent years [5]. Therefore, the way people deal with health issues is changing [1]. For example, it has been found that for pediatric consultations, mothers tend to use Internet resources frequently [6,7].

The delivery of health services using information and communication technologies (ICT), particularly the Internet, has been named eHealth, a concept that first appeared in 2000 [8]. Gunther Eysenbach published one of the most used definitions in 2001. This author defined eHealth as an emerging field at the intersection of medical informatics, public health, and business referring to the health services and information delivered or enhanced through the Internet and related technologies [9].

While Eysenbach’s eHealth definition seems to be the most accepted one, universal consensus does not exist [10]. There are essential eHealth aspects such as ICT [1], delivery of healthcare services [11], the Internet [10], and that it is user-centered [12], so eHealth can be understood to be the delivery of user-centered healthcare services through ICT, mainly the Internet.

Some distinctly important advantages are offered by eHealth. Numerous authors highlight its accessibility as one of its most relevant features [13,14]. It is important for users to access health information quickly and easily so they can resolve their queries [2,15,16]. A high degree of accessibility helps to overcome social and geographical barriers, allowing people with fewer resources to access health information and healthcare services [15,17,18]. Another important advantage is the possibility of tailoring interventions via eHealth [19], as personalized medical treatment can be more effective [20,21].

Users can be empowered by eHealth with regard to health issues [22]. This could help them make better informed health decisions [14,18] and aid in improving communication between people and healthcare providers [1], as eHealth is often used to supplement physicians’ recommendations [14,15]. Another advantage mentioned in the scientific literature is that eHealth allows people to access community support by facilitating participation in online support forums or in peer-support forums on social media [15,23].

The above notwithstanding, there are also some disadvantages to eHealth. For example, there are some serious concerns within the scientific community about the quality of the health information available online [24,25] as health-related web contents are not always trustworthy or validated [26,27,28]. Furthermore, information is not always easily understandable or suited to the needs of people [15,24,29]. Some authors have also described differences concerning the access to electronic health information as it relates to the digital divide, a concept that implies that socioeconomically disadvantaged subpopulations are less likely to have access to technologies, including eHealth interventions or health information available on the Internet [30,31]. Socioeconomically disadvantaged families also experience difficulty accessing technology or the Internet [32]. Some authors have described that the appearance of new medical technologies has often increased health disparities [33]. Technical issues could also become a barrier that can contribute to the digital divide [34].

The lack of education or training in the use of eHealth interventions could also generate personal barriers that can limit the access to health information [25]. Some authors state that eHealth can generate distrust among ordinary people. Numerous users are fearful of eHealth interventions and are reluctant to perform online health searches [24]. Parents in particular can feel unsafe and wary when searching health information [35]. Another disadvantage mentioned in the literature are the risk of adverse effects [2,27], especially in children [16]; concerns about privacy and security [34,36]; stress or anxiety of the users when performing health searches [37]; interference in the doctor–patient relationship [30]; or ethical and legal concerns [36].

Numerous authors propose some guiding principles for the future of eHealth. The principles most frequently mentioned in the literature are user empowerment and the improvement of their health and eHealth literacy [34,38]. In addition, healthcare providers should get involved in eHealth development and delivery [28]. It is also important to search for ways to minimize the digital divide [39] such as improving the usability of the eHealth interventions [25] and to investigate methods to ensure eHealth quality [10] and to develop ethical aspects [32].

The world of medicine and health cannot be understood without taking into account the social sciences. Social sciences cover such disciplines as psychology, education, management, public administration, communication, biomedical social sciences, social work, sociology, demography, information and documentation, legislation, etc. The strong focus on the detection and treatment of diseases has given way to a more holistic understanding of the patient, considering both purely medical and social aspects and placing the patient at the center of everything. Patienthood is a social state rather than simply a biological one. Thus, “psychosocial variables influence, not only the social and personal meanings of illness, but also the risk of becoming ill, the nature of the response to illness and its prognosis” [40].

The joint analysis of the social sciences and health allows professionals to understand not only medicine, but also the socioeconomic and political approach to disease and health. This interdisciplinary research facilitates different levels of analysis in the health sciences between social, psychological, behavioral, and biomedical scientists [41]. Thus, interdisciplinary efforts provide researchers new opportunities to refine theories and methods. Specifically, social scientists play different roles in health services, such as framing the issues, intelligence, monitoring, evaluation and assessment, and implementation, contributing to a better understanding of complex organizational arrangements, structures, cultures, management approaches, financial arrangements, and regulatory processes [42].

Social sciences have become an important approach in eHealth studies in the 21st century, and even more significantly in the last decade, a period in which the number of publications and citations has increased notably, as well as the number of areas of knowledge involved in these topics. The rapid and continuous development of new ICT has substantially changed the way in which people interact with healthcare systems [43]. Scholars have moved from debating what eHealth is to examining the technical, human, organizational, and social factors that influence eHealth practices [44,45,46]. Nowadays, eHealth research is an interdisciplinary field where information science and technology, biomedical science, and social sciences collaborate and create synergies [47].

To all of the above, the abrupt appearance of the coronavirus (COVID-19) pandemic during 2020 must also be added. As this pandemic requires quarantine and isolation, face-to-face visits in medical care have been considerably reduced. This situation calls for rapid and creative changes to the way healthcare is delivered and the development and adoption of new approaches to eHealth resources [48], which should be developed from a global vision, a vision which obviously must include the social sciences.

Despite the importance of this issue, there is a scarcity of systematic literature on what aspects of eHealth have been investigated from the perspective of social sciences. Although the existing bibliometric research addresses specific issues, it does not offer a holistic analysis of eHealth from the perspective of the social sciences. Along these lines there are some interesting papers to be found on topics such as health information systems [49]; Internet studies as a field of social science research around four primary research themes, including eHealth [50]; health informatics competences [51]; physical activity, sedentary behavior, and diet-related eHealth and mHealth [52]; international mobile health research [53]; or the most cited authors in a specific journal [54]. The two papers that carry out a more general analysis of these topics were written by Jiang et al. [41] who performed a systematic review of eHealth literature in the mainstream social science journals by testing the applicability of the 5A categorization (i.e., access, availability, appropriateness, acceptability, and applicability) and Son et al. [55] who reviewed the main research topics and trends of international eHealth through social network analysis.

The main objective of this research was to analyze the research on eHealth from the perspective of the social science areas of knowledge. To contextualize analysis of the relevant areas of knowledge of the documents analyzed, essential aspects like the number of publications per year, the most influential countries, and the most influential journals and papers are studied.

Therefore, this research contributes to academia by analyzing the state-of-the-art research on eHealth from the perspective of various social science areas of knowledge. It also identifies the main trends and proposes future lines of research and topics. To achieve this objective, a bibliometric analysis was developed. This paper has the following structure. First, the methodology is explained. Second, findings are presented to know the annual evolution of publications and citations, the most influential countries on these topics, the most relevant journals and papers, the most important areas of knowledge involved in this field, and significant trends. Finally, in conclusion, future lines of research are proposed.

## 2. Materials and Methods

For this study, a bibliometric analysis of the scientific literature in the Web of Science (WoS) Core Collection and a cluster analysis of the co-citation and keyword variables were carried out. The bibliometric analysis was based on the qualification and parameterization of scientific production as well as the influence of authors, publications, and institutions on a certain topic. The origin of this type of analysis is found in the article by Garfield [56] and his attempts to evaluate and quantify the importance of scientific articles. In 1960, he created the Institute for Scientific Information, which later became the WoS database. 

Bibliometry, as defined by Pritchard [57], is the application of mathematical and statistical methods to books and other communication methods. Therefore, and from this perspective, bibliometric analysis is a meta-analytic systematic review. The success of this methodology lies in the possibility of measuring scientific activity to quickly and concisely study the antecedents, evolution, trends, and future lines of research of a topic, measuring scientific activity around a given topic.

The impact or influence is measured by the number of citations an article receives. In an attempt to unify both positions, Hirsch [58] created an index that provides a balance between the number of articles and citations (h-index).

The procedure used for data collection and subsequent information analysis has been described by Moed [59] or Brereton et al. [60], although there are multiple variants to these procedures. The first stage consisted of selecting the WoS Core Collection database, a source that has been commonly used in bibliometric analysis. It was the first compiler of indexes and a precursor in measuring the impact of journals and covers more research fields compared to other databases. In addition, WoS allows filtering the indicators, prioritization by number of citations, and its journal impact index guarantees the quality of articles.

Five search terms were chosen based on the prevailing literature on the topic: eHealth; mHealth; Telemedicine; Mobile Health; and Telehealth. The documents published in 2020 were eliminated, as the year had not finished at the time of this study and their inclusion could distort the analysis. Furthermore, other documents such as grey literature, books, or proceedings were excluded, limiting the search to the articles published in indexed journals.

**Figure 1:**
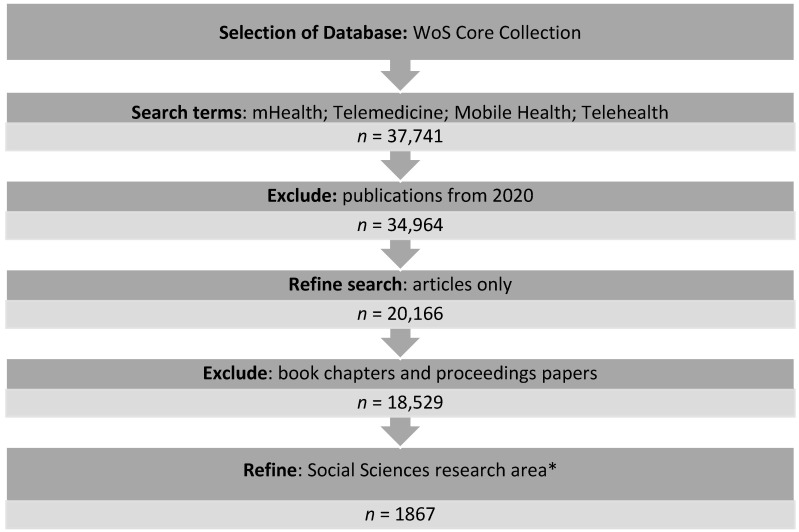
Methodology wtages used in the bibliometric analysis. * Archaeology; Area Studies; Biomedical Social Sciences; Business & Economics; Communication; Criminology & Penology; Cultural Studies; Demography; Development Studies; Education & Educational Research; Ethnic Studies; Family Studies; Geography; Government & Law; International Relations; Linguistics; Mathematical Methods In Social Sciences; Psychology; Public Administration; Social Issues; Social Sciences—Other Topics; Social Work; Sociology; Urban Studies; Women’s Studies.

The documents in the WoS database are classified into five broad categories: Arts and Humanities; Life Sciences and Biomedicine; Physical Sciences; Social Sciences; and Technology, with all the journals assigned to at least one research area. The final research criterion used was to refine the search by the research related to social sciences (Figure 1).

Once the data had been cleaned, the results were exported to files compatible with statistical analysis tools, performing a cluster analysis through the VOSviewer [61]. The text mining functionality of this tool supports the generation of keyword term maps based on a corpus of documents [62]. A term map is a two-dimensional map in which words are located in such a way that the distance between them can be taken as an indication of the affinity of the terms. The relatedness of terms is determined by their cooccurrence in documents [63].

The analysis was limited to the terms that were repeated a minimum of 25 times (111 keywords) with the keywords used for the search eliminated from the count. In this analysis, keywords from authors, journals, as well as the most repeated words in titles and abstracts were selected. 

This study also used fractional counting at the network level since it can normalize the relative weights of links and thereby clarify structures in the network [64].

## 3. Results and Discussion

### 3.1. Publications Per Year

The first article to focus on the eHealth topic included in the WoS database in the Social Sciences research area is “Some implications of Telemedicine” by Ben Park and Rashid Bashshur published in 1975 in *Journal of Communication* [65]. This paper, published before the existence of the Internet, prophesied that healthcare delivery by two-way television might change roles, authority, and distribution of healthcare professionals. 

The number of scientific publications on eHealth during the 20th century is small, even in the late 1990s when mobile phones and the Internet were in common use. It was not until the decade of 2010 when there was an important increase with the number of publications doubling from 199 to 433 (Table 1). Since 2005, there has been a continuous annual growing of manuscripts, with 2019 having the largest number of publications (317).

**Figure 2:**
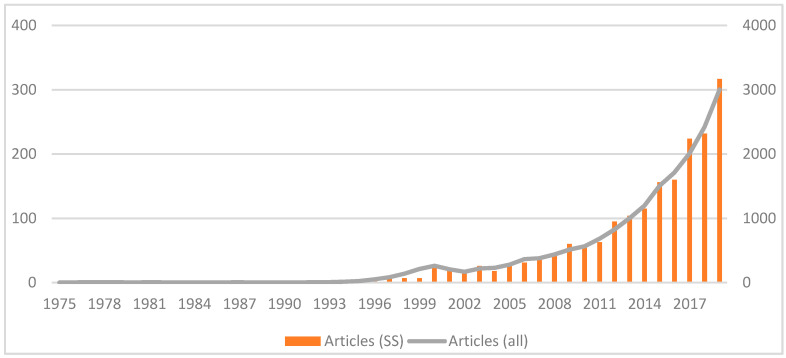
Evolution in the number of articles (Social Sciences (SS) and all areas).

The comparison between articles including all research areas and those limited to the Social Sciences (Figure 2) shows a similar evolution. The lack of differences confirm that the topic is developing in the same way across the whole scientific community. This parallel evolution does not happen when the field does not generate significant scientific interest. 

### 3.2. Most Influential Countries

The ten countries with the largest number of articles published related to eHealth in Social Sciences are shown in Table 2. The USA is the country with the largest number of articles published and citations, with 7.38 times more articles than the second-ranking country, Australia. Among the rest of the countries shown in the table, two groups can be distinguished: Australia, the UK, and Canada have a similar number or articles, between 104 and 150, while the remaining countries (Netherlands, Germany, Spain, China, Italy, South Africa) have a smaller number of publications, between 36 and 71.

When analyzing the number of citations, the USA is once again the highest-ranking country, 6.65 times higher than the second country, the UK. Nevertheless, the h-index of the USA is only 1.96 times higher than that of the UK. If we consider mean citations per article, the largest number corresponds to China, with a mean of 71.24 citations per article. This figure seems very high, as it is 3.47 times higher than mean citations of the second most cited country, the UK, considering that China has only 37 articles compared to the 3038 articles published in the UK.

Only three of the ten countries (USA, China, and UK) have eleven articles with more than 250 citations. It is important to highlight that although the USA and China have a similar number of articles in this category, the number of articles published in the USA (1107) far outnumbers the 37 articles published in China. In addition, when considering the categories with more than 100, 50, and 25 citations, China has larger figures than expected when considering the number of articles published and the h-index of each country. Perhaps, this particular finding could benefit from a more detailed analysis of the Chinese articles to find how they are cited and interconnected. If Chinese articles are not taken in account, the rest of the figures of these rankings are in the same order as the list of countries with more published articles.

Another aspect to consider when analyzing the most influential countries is the number of citations in relation to the population of each country (Table 3). In this case, the country with the largest number of citations per population is Australia, followed by the Netherlands, the USA, the UK, and Canada. Despite the large number of absolute citations and the large number of citations per article, China is in the last place due to its large population.

It seems understandable that a country like the USA has the largest number of publications due to its large population, but surprisingly this is not the case for China, perhaps because their literature production about eHealth is less focused on social sciences. Analyzing the rest of the list, we can find countries like Australia, the UK, Canada, the Netherlands, or Germany, which seem to be more concerned with the development of the social sciences literature.

### 3.3. Most Influential Journals and Papers

When analyzing the most influential journals related to eHealth in the social sciences, the number of articles published on these topics and the number of citations have been taken into account. The results of the said analysis can be seen in Table 4 in the ranking of the most influential journals. The ranking is led by the journal *Professional Psychology Research and Practice* with 54 articles and 1768 citations in addition to having the highest h-index (23). This journal is followed by *Patient Education and Counseling* (a medical journal covering patient education and health communication) with 44 articles and 880 citations and by *Journal of Health Communication* (focused on information and library science), 42 articles and 1026 citations. However, the high impact of the journal *Social Science & Medicine* is very striking since, with 24 articles on this topic, it has received 1043 citations, which makes it the journal with the largest number of citations per article (43.46).

**Figure 3:**
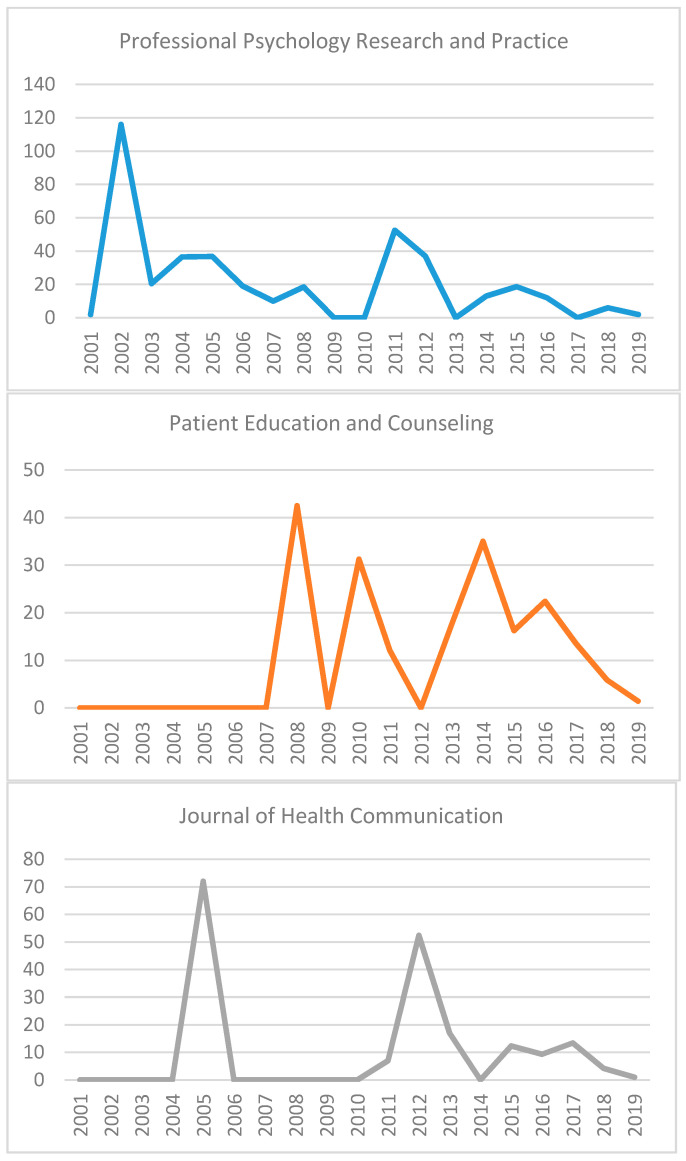

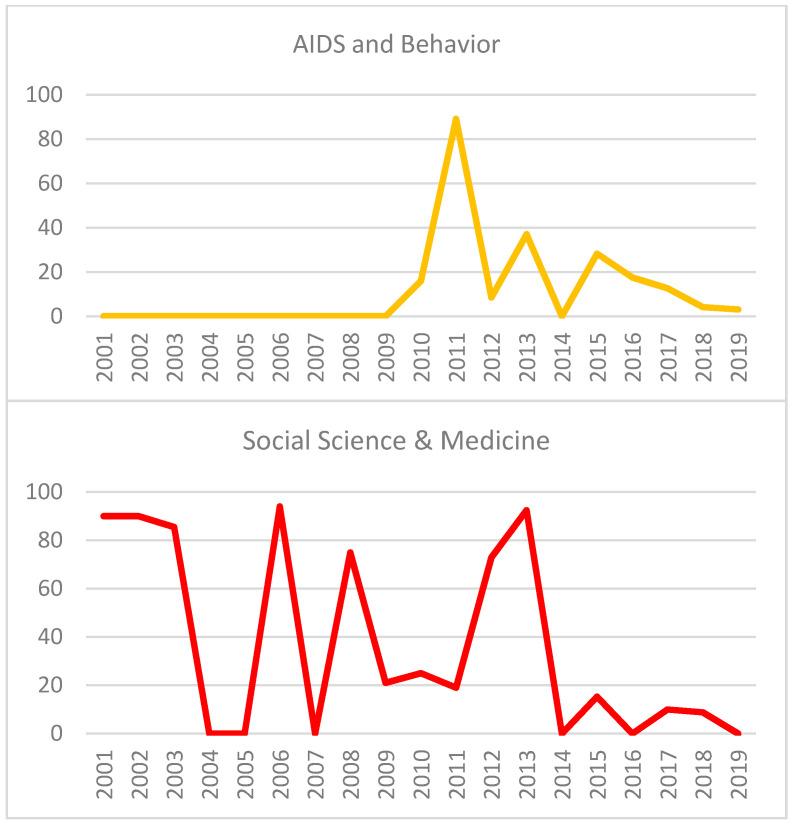
Citations per article of journals in the 21st century.

When focusing the analysis on the articles published in the 21st century, which represent 96.52% of the total articles on these topics, it can be observed (Figure 3) that the most relevant journals are *Social Science & Medicine* (43.26), *Professional Psychology Research and Practice* (28.53), *Journal of Health Communication* (24.43), *Patient Education and Counseling* (19.33), and *AIDS and Behavior* (13.71).

It is noteworthy that, of the top ten journals that publish articles about eHealth in the field of Social Sciences, 50% of them have psychology applied to various fields as their main field of research. This is the case for *Professional Psychology Research and Practice* (psychology, multidisciplinary), *Psychological Services* (psychology, clinical), Psycho-Oncology (psychological aspects of oncology), *Frontiers in Psychology* (psychology, multidisciplinary), and *Journal of Pediatric Psychology* (child psychology).

**Figure 4:**
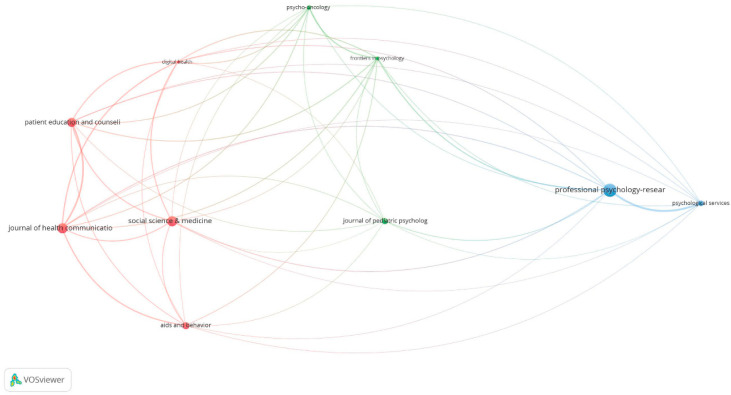
Cluster analysis of co-citations among the most relevant journals.

Figure 4 shows a cluster analysis of co-citations among the most relevant journals in this field of research. This analysis is based on the existence of thematic similarity between two or more documents that are co-cited in a third and subsequent work. Thus, the higher the frequency of co-citation, the greater the affinity between them. Three main clusters were identified. Two of them are directly related to aspects of psychology led *by Professional Psychology Research and Practice* and *Journal of Pediatric Psychology*. The other cluster is more focused on health and medicine, with a central axis in the journal *Social Science & Medicine*, which has close relationships with *Journal of Health Communication* and with *Patient Education and Counseling* among others.

With regard to the articles with the largest number of citations (Table 5), three of the top ten were published in *Information & Management*, a journal mainly focused on the field of information systems and applications which, in this case, are focused on eHealth. The four articles with the most citations have a common central element, the analysis of the technology acceptance model (TAM). The first article, “Why do people play on-line games? An extended TAM with social influences and flow experience” [67] analyzes the reasons why people play online games using the TAM model, connecting social influence, psychology, and telemedicine technology (778 citations). The second article (with 756 citations), “Examining the technology acceptance model using physician acceptance of telemedicine technology” [68], studies the applicability of the TAM model for explaining physicians’ decisions for accepting telemedicine technology in the healthcare context, providing some implications for user technology acceptance research and telemedicine management. The third article, with 548 citations, “Information technology acceptance by individual professionals: A model comparison approach” [69] represents a conceptual replication of several model comparison studies, TAM, theory of planned behavior (TPB), and a deconstructed TPB model, by analyzing the responses to a survey on telemedicine technology acceptance. The fourth article, “Investigating healthcare professionals’ decisions to accept telemedicine technology: an empirical test of competing theories” [70], has 425 citations and evaluates the extent to which prevailing intention-based models, including TAM, TPB, and an integrated model, could explain physicians’ acceptance of telemedicine technology.

Another featured article is “mHealth for Mental Health: Integrating Smartphone Technology in Behavioral Healthcare” [71], which provides an overview of smartphone use in behavioral healthcare and discusses options for integrating mobile technology into clinical practice (375 citations; 4688 citations per year). The article “Interdisciplinary Chronic Pain Management Past, Present, and Future” [72], with 215 citations, is the third document with a large number of citations per year (43). This research discussed the major components of a true interdisciplinary pain management program, providing future directions in this field, including telehealth.

### 3.4. Relevant Areas of Knowledge

Given that eHealth is an issue that cuts across many disciplines, it is not surprising that research on this issue is of interest to researchers in numerous fields and involves many areas of knowledge within the social sciences. Among these knowledge areas, Psychology is the most relevant, with 778 articles published on this topic and 14,158 citations, having an h-index of 54 (Table 6). This corresponds to the findings on the most relevant journals since, as previously stated, half of those in the top ten have psychology as applied to various fields as their main field of research. Thus, psychology becomes the human dimension of digital health. The future of psychology should be conducted through technology and patient empowerment. Patient social networks are becoming an important instrument for empowering patients and their families in managing their disease. Thus, one of the challenges faced by eHealth with online interventions is for people to change their attitude and/or their behavior. Among the many articles of Psychology on this topic, there are 19 that have more than 100 citations, two of which even exceed 250 citations: “mHealth for Mental Health: Integrating Smartphone Technology in Behavioral Healthcare” [69] and “A Behavior Change Model for Internet Interventions” [74]. 

Other areas which play a prominent role in research on this topic are *Education & Educational Research* (248 articles); *Biomedical Social Sciences* (234); Business & Economics (189); *Social Sciences—Other Topics* (170); and *Communication* (135). In relation to *Education & Educational Research*, it is observed that medical care has evolved from more disease-focused care to patient-directed care, including in the field of health education. The works published in this area mainly investigate aspects related to the design, implementation and evaluation of eHealth education. The aim is to empower health professionals and the general public in terms of health education and digital skills, to promote healthy lifestyle habits and achieve a more active and participatory role in relation to individual and community health and well-being. The article with the most citations (168) in this field is entitled “Internet use for health information among college students” [77].

Of import within the field of *Biomedical Social Sciences* is the development of methods of analysis and processing of biomedical signals and images to aid the diagnosis of different pathologies, as well as the generation of predictive models based on bio-signals and symptoms with applications in the field of eHealth. “Quantifying the body: monitoring and measuring health in the age of mHealth technologies” [78] is the paper with the largest number of citations in this area (189). 

While the Business & Economics area ranks fourth in terms of the number of articles published, this field has the largest average number of citations per paper (31.28), which shows the interest of academia in this topic. In fact, the paper with the largest number of citations on this topic is precisely from the Business & Economics area, the aforementioned work by Hsu and Lu [67] “Why do people play on-line games? An extended TAM with social influences and flow experience”. Another of the great challenges of research in this field is an efficient integration of eHealth within public systems, with special focus on the reduction of costs and, at the same time, of patient waiting times.

**Figure 5:**
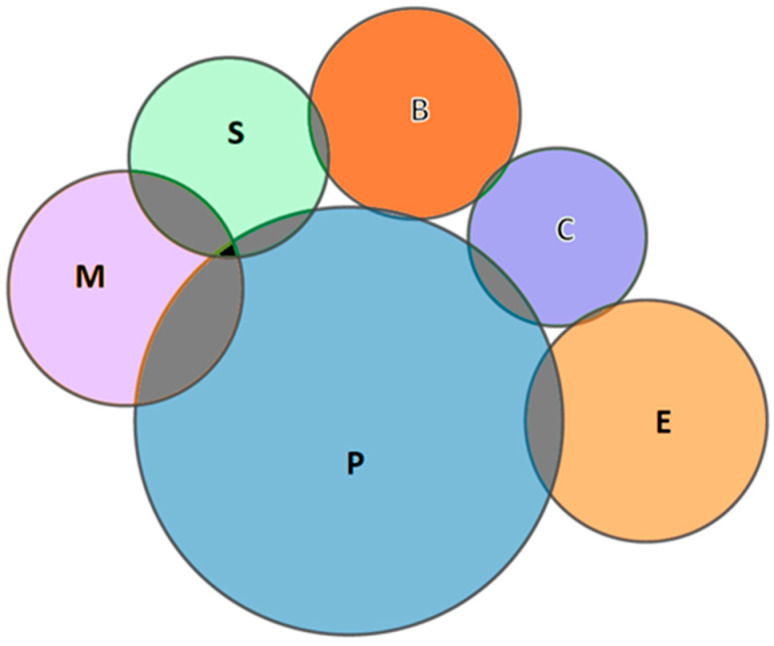
Venn diagram of the interrelations between areas of knowledge. P: Psychology; E: Education & Educational Research; M: Biomedical Social Sciences; B: Business & Economics; S: Social Sciences—Other Topics; C: Communication.

Another aspect to consider is the interrelation between the areas of knowledge, that is, papers related to social sciences and medicine that are framed in more than one area at the same time. For this, a Venn diagram was used, considering the six research areas with more than 100 papers published in this field (Figure 5). Once again, it can be seen that Psychology plays the central role as it is linked with the other five areas, highlighting its close relationship with Biomedical Social Sciences, sharing 51 papers, and with Education & Educational Research (20). Psychology shares other papers with Social Sciences—Other Topics (10), Communication (10), and Business (1).

Furthermore, the Social Sciences—Other Topics area, given its transversal nature, shares research with other fields, such as Biomedical Social Sciences (26), and Business & Economics (4). Specifically, there is a paper by Fraser [79] published in International Journal of Transgenderism, which is framed within three different research areas: Psychology, Biomedical Social Sciences, and Social Sciences—Other Topics.

### 3.5. Keywords and Trends

The analysis found 105 keyword terms that appeared a minimum of 25 times. It seems logical that the most used terms are “care”, “technology”, “Internet”, and “health”. It is noteworthy that the fifth most used term is “depression”, a finding that seems consistent with the fact that Psychology was the most relevant area of knowledge found in the analysis. On the other hand, despite Education & Educational Research being the second most relevant area, the first term related with this area, “education”, was ranked 15th.

**Figure 6:**
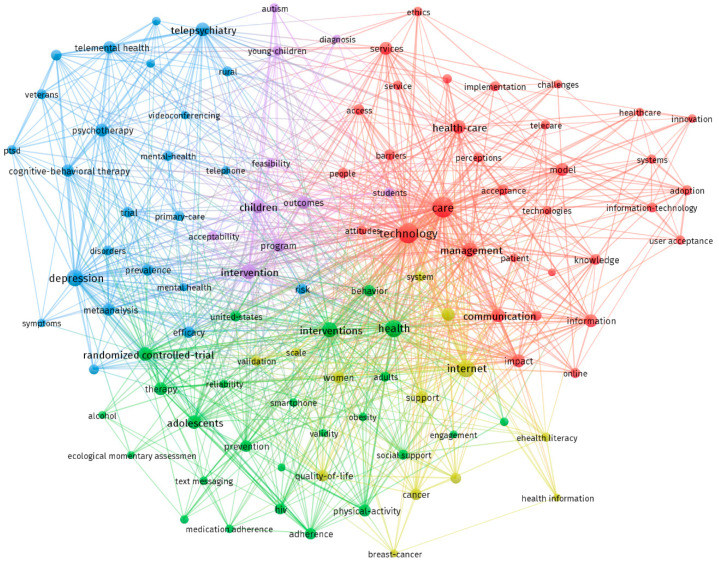
Cluster visualization of co-occurrence of keywords.

The analysis of the terms showed five clearly identified clusters (Figure 6). The cluster in red color is focused on the nuclear terms related to eHealth, with keywords that define the concept, like “information”, “communication”, “management”, “technology”, “online”, or “digital health”. The technology and innovation features of eHealth are also represented by keywords like “implementation”, “innovation”, “services”, “system”, or “technology”, as these are essential aspects of the very concept of eHealth. Other important keywords found were “challenges”, “barriers”, or “ethics”, which reflect some of the problems that the eHealth can deliver. Finally, one of the most important aspects of eHealth, the users, is featured in this cluster with terms like “patient” or “people”, but also with “attitude”, “perceptions”, “satisfaction”, or “user acceptance”.

The cluster in green is focused on three aspects related with the social features of the use of eHealth. Keywords like “adolescents”, “adults”, “behavior”, “behavior-change”, “engagement”, “smartphone”, or “self-efficacy” are related with aspects of the users that use eHealth interventions. Keywords like “alcohol”, “health”, “HIV”, “obesity”, “physical activity”, or “prevention” reflect the medical aspects that concern people. Finally, keywords like “smartphone”, “social support”, or “text messaging” reflect how eHealth has the potential to allow people to access community support.

The cluster in blue highlights the importance of psychological and mental health aspects in this field, grouping keywords like “anxiety”, “depression”, “mental health”, “psychotherapy”, or “telepsychiatry”. This seems logical as Psychology is the most relevant area of knowledge found in the analysis, reflecting that this field is an important part of the eHealth literature when analyzed from the point of view of the social sciences.

The cluster in yellow reflects two related aspects, women and health literacy, as women use more eHealth and have more health and eHealth literacy. Finally, the fifth cluster (purple color) is related to children, with keywords like “autism”, “children”, “students”, and “young children”.

A trend analysis showed that some of these terms currently being used most frequently are “acceptance”, “acceptability”, “engagement”, “eHealth literacy”, or “barriers”. As has been found in the literature, this seems to confirm that the main guidelines for future research concern acceptance, increasing eHealth literacy of users, and overcoming barriers.

## 4. Conclusions

Social sciences play an increasingly important role in eHealth. From the information obtained, the time-based progression of the number of articles published is particularly significant, showing the interest of the scientific community in this topic and a constant increase in research works. The USA is the country with the largest number of published articles and citations. China has the largest mean number of citations per article, although the highest h-index belongs to the USA. Only three of the ten countries (USA, China, and UK) have 11 articles with more than 250 citations. Finally, Australia is the country with most citations considering the population of the country.

With regard to the number of articles and the h-index, Professional Psychology Research and Practice is the most influential journal on eHealth in Social Sciences, followed by Patient Education and Counseling and Journal of Health Communication. However, Social Science & Medicine has the largest number of citations per article. A cluster analysis of co-citations in the most relevant journals identified three main clusters. Two of them are focused on different aspects of Psychology, which is very significant since 50% of the most relevant journals in this field are closely related to this area of knowledge. The other cluster is directly related to Health and Medicine. Most (96.52%) of the articles on these topics have been published in the 21st century. The analysis of the TAM is the central axis of some of the most cited articles. Nevertheless, there are other subjects of great interest, such as the information systems field oriented to eHealth, the use of smartphones in behavioral healthcare, the applications for integrating mobile technology into clinical practice, or an interdisciplinary pain management program in eHealth.

It is notable that the relationship of patients with the health system has changed. The concept of the passive patient has fallen by the wayside in favor of people who are more active and involved in all processes. As a result, eHealth is a very transversal field for the different areas of social sciences. Although there are many areas of knowledge and different fields of Social Sciences related to research on eHealth, Psychology stands out above all others. One of the important research trends in this field will continue to be the empowerment of patients (and people in general) through technology, as well as helping change people’s attitudes and behaviors, based on psychological theories and principles. This will offer new opportunities for both theoretical and applied research.

Other relevant areas in this field are Education & Educational Research; Biomedical Social Sciences; Business & Economics; Social Sciences—Other Topics; and Communication. Education & Educational Research is focused on the design, implementation, and evaluation of eHealth education. Based on the findings of this research, it appears that in the future, there will be a growing interest in the acquisition of knowledge at different levels related to both health education and digital skills addressed to different groups, both medical professionals and people in general (particularly, in certain targeted population groups, such as elderly or ethnic groups). 

The potential of eHealth as a professional training instrument will improve the quality of care provided to the population, as well as develop new sources of knowledge and research. In Biomedical Social Sciences, there are still good opportunities for research with regard to the methods of processing biomedical signals and the development of predictive models in eHealth. Business & Economics is the area with the largest average number of citations per paper. One of the challenges of research in this field is the analysis of the eHealth TAM (as well as the extended version), including cultural and social factors, to empirically assess the validity of its constructs, mainly its level of helpfulness, usability, and intention to use eHealth services. Other important research lines are the efficient integration of eHealth within public systems, efficient budget management, or the improvement in the quality of service for patients, and improved perception by all stakeholders. In addition, social sciences have tools to measure different types of outcomes.

Furthermore, it is important to highlight the interaction between the different areas of knowledge. Once again, Psychology plays a central role, sharing research with the other most relevant areas, mainly with Biomedical Social Sciences and Education & Educational Research. For future research, it would be necessary to promote even more synergy between different disciplines.

The most used terms were grouped into five main clusters focused on nuclear terms related to eHealth are “care”, “technology”, “Internet”, and “health”; aspects related to social features and the use of eHealth; and psychological and mental health aspects in this field. The main trends found are studying acceptability, increasing eHealth literacy of users, and overcoming barriers.

This work is not exempt from some limitations, some of which could be the basis for future research. Thus, in addition to the use of WoS, other quantitative and/or qualitative tools could also be utilized. Finally, other terms related to eHealth, including broader concepts, could be analyzed.

## Figures and Tables

**Table 1:** Number of articles per year.

Years	Articles	Citations	h-Index	Mean	≥100	≥50	≥25	≥10
2015–19	1103	8475	35	7.68	6	19	70	256
2010–14	433	11,096	53	25.63	15	60	140	279
2005–09	199	5962	44	29.96	7	39	85	125
2000–04	109	4735	33	43.44	6	20	45	72
1995–99	29	1575	16	54.31	3	6	11	16
1990–94	0	0	0	0.00	0	0	0	0
1985–89	1	10	1	10.00	0	0	0	1
1980–84	1	6	1	6.00	0	0	0	0
1975–79	6	59	4	9.83	0	0	1	2
Total	1881	31,918	73	16.97	37	144	352	751

**Table 2:** Number of articles and citations by country.

Country	A	C	h	Mean	≥250 C	≥100 C	≥50 C	≥25 C	≥10 C	≥5 C	≥1 C
USA	1107	20,229	59	18.27	6	22	92	228	485	687	1026
Australia	150	2913	29	19.42	0	5	17	35	65	87	136
UK	148	3038	30	20.53	1	5	16	36	64	82	132
Canada	104	1475	20	14.18	0	1	4	17	44	61	91
Netherlands	71	1347	22	18.97	0	1	7	20	34	45	61
Germany	50	557	11	11.14	0	1	2	6	13	23	42
Spain	41	448	10	10.93	0	1	3	5	11	15	30
China	37	2636	14	71.24	4	6	7	10	16	21	32
Italy	36	370	10	10,28	0	0	1	5	10	20	32
South Africa	36	186	8	5,17	0	0	0	1	5	12	28

A: articles; C: citations; h: h-index.

**Table 3:** Mean citations per population.

Country	Population *	Citations	Mean
Australia	25,499,884	2913	0.114236
Netherlands	17,134,872	1347	0.000079
USA	331,002,651	20,229	0.000061
UK	56,286,961	3038	0.000054
Canada	37,742,154	1475	0.000039
Spain	46,754,778	448	0.000010
Germany	83,783,942	557	0.000007
Italy	60,461,826	370	0.000006
South Africa	59,308,690	186	0.000003
China	1,439,323,776	2636	0.000002

* Source of population data: United Nations 2020 [66].

**Table 4:** Most relevant journals on the eHealth and Social Sciences.

Journal	Articles	Citations	h-Index	Cit/Paper	IF-5 Years	Q
Professional Psychology Research and Practice	54	1768	23	32.74	2.077	Q2
Patient Education and Counseling	44	880	18	20	3.408	Q1
Journal of Health Communication	42	1026	17	24.26	2.358	Q2
Digital Health	39	106	5	2.72	-	-
AIDS and Behavior	38	520	14	13.68	3.298	Q1
Psychological Services	33	335	10	10.15	2.201	Q2
Psycho-Oncology	31	215	7	6.94	3.581	Q1
Frontiers in Psychology	24	147	7	5.92	2.723	Q2
Social Science & Medicine	24	1043	15	43.46	4.241	Q1
Journal of Pediatric Psychology	23	384	11	16.7	3.505	Q3

Cit/paper: citations per paper; IF-5 years: impact factor in the last five years; Q: quartile in WoS.

**Table 5:** Articles with the largest number of citations on eHealth and social sciences.

R	Article	C	Journal	Reference	C/Y
1	Why do people play on-line games? An extended TAM with social influences and flow experience	778	Information & Management	Hsu, C.L.; Lu, H.P. (2004) [67]	51.87
2	Examining the technology acceptance model using physician acceptance of telemedicine technology	756	Journal of Management Information Systems	Hu, P.J.; Chau, P.Y.K.; Sheng, O.R.L.; Tam, K.Y. (1999) [68]	37.80
3	Information technology acceptance by individual professionals: A model comparison approach	548	Decision Sciences	Chau, P.Y.K.; Hu, P.J.H. (2001) [69]	30.44
4	Investigating healthcare professionals’ decisions to accept telemedicine technology: an empirical test of competing theories	425	Information & Management	Chau, P.Y.K.; Hu, P.J.H. (2002) [70]	25.00
5	mHealth for Mental Health: Integrating Smartphone Technology in Behavioral Healthcare	375	Professional Psychology-Research and Practice	Luxton, D.D.; McCann, R.A.; Bush, N.E.; Mishkind, M.C.; Reger, G.M. (2011) [71]	46.88
6	Zooming In and Out: Studying Practices by Switching Theoretical Lenses and Trailing Connections	273	Organization Studies	Nicolini, D. (2009) [73]	27.30
7	A Behavior Change Model for Internet Interventions	266	Annals of Behavioral Medicine	Ritterband, L.M.; Thorndike, F.P.; Cox, D.J.; Kovatchev, B.P.; Gonder-Frederick, L.A. (2009) [74]	26.60
8	Examining a model of information technology acceptance by individual professionals: An exploratory study	259	Journal of Management Information Systems	Chau, P.Y.K.; Hu, P.J. (2002) [75]	15.24
9	Interdisciplinary Chronic Pain Management Past, Present, and Future	215	American Psychologist	Gatchel, R.J.; McGeary, D.D.; McGeary, C.A.; Lippe, B. (2014) [72]	43.00
10	Technology acceptance model for internet banking: an invariance analysis	208	Information & Management	Lai, V.S.; Li, H.L. (2005) [76]	14.86

R: rank; C: total citations; C/Y: citations per year.

**Table 6:** Relevance of areas of knowledge on eHealth and social sciences.

Area of Knowledge	Articles	Citations	h-Index	Average
Psychology	778	14,158	54	18.20
Education & Educational Research	248	2513	25	10.13
Biomedical Social Sciences	234	3878	34	16.57
Business & Economics	189	5911	32	31.28
Social Sciences—Other Topics	170	2010	25	11.82
Communication	135	2207	26	16.35
Social Work	59	482	13	8.17
Government & Law	57	364	9	6.39
Linguistics	52	888	18	17.08
Family Studies	40	431	13	10.78
Social Issues	31	486	11	15.68
Sociology	25	756	12	30.24
Public Administration	19	195	7	10.26
Women’s Studies	16	161	6	10.06
Development Studies	15	68	5	4.53
Criminology & Penology	12	71	5	5.92
Geography	10	110	5	11.00
International Relations	4	16	2	4.00
Urban Studies	4	6	1	1.50
Area Studies	3	6	2	2.00
Ethnic Studies	3	51	2	17.00
Demography	1	3	1	3.00

## Data Availability

The data presented in this study are available on request from the corresponding author.
